# Super-resolution observation of lysosomal dynamics with fluorescent gold nanoparticles

**DOI:** 10.7150/thno.42134

**Published:** 2020-05-15

**Authors:** Kangqiang Qiu, Yang Du, Jiyan Liu, Jun-Lin Guan, Hui Chao, Jiajie Diao

**Affiliations:** 1 MOE Key Laboratory of Bioinorganic and Synthetic Chemistry, School of Chemistry, Sun Yat-Sen University, Guangzhou 510275, China.; 2 Department of Cancer Biology, University of Cincinnati College of Medicine, Cincinnati, OH 45267, USA.; 3 Department of Biotherapy, Cancer Center, State Key Laboratory of Biotherapy, West China Hospital, West China Medical School, Sichuan University, Chengdu 610041, China.

**Keywords:** super-resolution imaging, structured illumination microscopy, lysosomes, mitophagy, long-term labelling

## Abstract

Because lysosomes play critical roles in multiple cellular functions and are associated with many diseases, studying them at the subcellular level could elucidate their functionality and support the discovery of therapeutic drugs for treating those diseases. The commonly used dyes for super-resolution imaging of lysosomes are the commercial molecular LysoTrackers. But the tolerance to changes in the lysosomal microenvironment and to lysosomal membrane permeabilization (LMP) and the photostability of the LysoTrackers are worrisome. The purpose of our study was to evaluate the feasibility of performing a fluorescent gold nanoprobe for super-resolution observation of lysosomal dynamics in living cells and compare it to the commercial LysoTrackers.

**Methods**: The nanoprobe **Cy5@Au NP** contained three parts: a bio-inert gold core, a biocompatible polyethylene glycol spacer, and a fluorophore cyanine 5. Structured illumination microscopy (SIM) was employed to capture the fluorescence of **Cy5@Au NPs** in cells. The tolerance assays to changes in the lysosomal microenvironment and to LMP, the photobleaching assay, and the long-term lysosomes labelling assay of **Cy5@Au NPs** were compared with commercial LysoTrackers. The super-resolution observation of lysosomal dynamics with **Cy5@Au NPs** was performed.

**Results**: **Cy5@Au NPs** can light up lysosomes specifically under SIM. Compared with commercial lysosomal molecular probes, **Cy5@Au NPs** exhibited stronger tolerance in lysosomes during various treatments, and changes in the lysosomal microenvironment and LMP did not cause **Cy5@Au NPs** to lose track of their targets. **Cy5@Au NPs** demonstrated an excellent anti-photobleaching ability, and a long-term labelling assay revealed that they could label lysosomes more than 3 d. Biological events of lysosomes such as the kiss-and-run process, fusion, fission, and mitophagy were recorded with the fluorescent **Cy5@Au NPs** under SIM.

**Conclusions**: The nanoprobe **Cy5@Au NP** was successfully used as a lysosomal probe for the super-resolution observation in living cells and found to overcome the limitations of commercial LysoTrackers. Our results thus confirm that nanoparticles can be useful tools for subcellular super-resolution imaging and highlight new avenues for using nanoparticles in biology.

## Introduction

As the basic components of cells, organelles play important roles in cellular processes and interact with each other in various ways 1-5. In living cells, organelles have often been visualized by using noninvasive fluorescence microscopy 6-8. Conventional fluorescence imaging technology is subject to an optical diffraction limit, however, and thus cannot sufficiently resolve the spatial resolution beyond 200 nm, which restricts its applicability in distinguishing the interplay between subcellular structures 9, 10. More recently, with the rapid development of super-resolution technology, many types of super-resolution microscopy—stochastic optical reconstruction microscopy (STORM), stimulated emission depletion (STED) microscopy, and structured illumination microscopy (SIM), for example—have overcome the diffraction limit (below 200 nm) of conventional optical microscopes and achieved super-resolution imaging at the subcellular level 11-16. Among those techniques, SIM excites samples by using patterned illumination, with a low illumination intensity and short acquisition time for imaging that risk only slight photodamage to cells 17-22. Though the spatial resolution of SIM microscopy (100-120 nm) is not at the same level as what can be achieved by STED or STORM techniques (tens of nanometer), it is more than sufficient for investigating organelles. For all of these reasons, SIM can be especially useful for investigating subcellular dynamics in living cells.

Acidic organelles with a variety of acid hydrolases, lysosomes are coated with a phospholipid bilayer in cells 23. Along with their well-known function as cellular recycling centers that degrade biomacromolecules from endocytosis, autophagy, and phagocytosis 24-26, lysosomes also play critical roles in a range of cellular functions, including plasma membrane repair, cholesterol homeostasis, energy homeostasis, the activation of apoptosis, the generation of building blocks for cell growth, cell migration, activating transcriptional programs, and priming tissues for angiogenesis and metastasis formation 27-29. As a consequence, the malfunction of lysosomes can cause diseases such as silicosis, cancer, cardiovascular disorders, Parkinson's disease, lysosomal storage diseases, immune system disorders, and neurodegenerative disorders 30-32. At the same time, most of those lysosomal functions and associated diseases have themselves been found to be associated with other organelles 2-4. For example, lysosome-mitochondrion interaction is associated with the cellular function of autophagy, along with diseases such as cancer as well as neurodegenerative disorders 33, 34. Therefore, studying lysosomes at the subcellular level stands to shed light on their functionality and associations with diseases and, in turn, aid the discovery of new therapeutic drugs for such diseases.

To visualize lysosomes, many tags—organic molecules, metal complexes, proteins, and quantum dots, for instance—have been applied in fluorescence imaging 35-40. Although few such dyes are applicable with SIM imaging, the two most widely used ones are commercial lysosomal dyes: LysoTracker Green (LTG) and LysoTracker Red (LTR) 8, 9, 17, 19. Those commercial dyes are small molecules that target lysosomes according to their acidic pH, which helps to protonate weak base groups on dyes for trapping lysosomes, thereby achieving the recognition of lysosomes themselves 41, 42. However, because the pH of lysosomes is unstable and changes when stimulated by drugs (e.g., chloroquine), these commercial dyes can easily become unspecific to lysosomes 43, 44. Beyond that, common treatments such as cell fixation can cause lysosomal membrane permeabilization (LMP), thereby allowing commercial molecular probes to escape from lysosomes 45, 46. For that reason, it is important to develop lysosomal probes that are applicable to a variety of situations for SIM imaging.

To overcome the limit of small molecules during LMP, nanoparticles were used as lysosomal probes in our study. Due to their larger size, nanoparticles have a diffusion limit during LMP, and a natural slow exocytosis, which prolongs their retention time in lysosomes. To localize in lysosomes specifically, modification with lysosomal targeting groups is useful, and applies equally to nanoparticles. Meanwhile, most commonly, nanoparticles can entrap into endosomes and subsequently lysosomes by endocytosis process, leading to no requirement for a further derivation with targeting group 47-52. Herein, with bio-inert gold nanoparticles as the core, biocompatible polyethylene glycol (PEG) was modified on the surface, and a fluorophore cyanine 5 (Cy5) for distinguishing the common colors of commercial dyes was coupled to the PEG terminal end in order to obtain the fluorescent **Cy5@Au NPs**. Able to localize in the lysosome, the particles can overcome the limitations of commercial lysosomal probes, and under SIM illumination, lysosomal events such as kiss-and-run process, fusion, fission, and mitophagy can be observed.

## Materials and Methods

### Materials

Carbonyl cyanide *m*-chlorophenylhydrazone (CCCP, #C2759) was purchased from Sigma, and MitoTracker Green FM (MTG, #M7514), LTR (#L7528), and LTG (#L7526) were purchased from Invitrogen (Thermo Fisher Scientific, USA). Penicillin-streptomycin (#15140163, 10,000 units/mL), fetal bovine serum (FBS, #26140079), and Dulbecco's modified Eagle's medium (DMEM, #11965092) were all purchased from Gibco (Thermo Fisher Scientific, USA). Phosphate-buffered saline (PBS, #SH30256.01) was purchased from Hyclone (GE Healthcare Life Sciences) and the autophagosome detection dye (DAPGreen, #D676) from Dojindo Molecular Technologies, Inc (Japan). LAMP1-mGFP was obtained from Addgene (plasmid, #34831) 53. The fluorescent gold nanoparticles were custom synthesized by Luna Nanotech Inc. (Toronto, Canada); the size of the gold core was 15 nm, with 5 kDa of PEG and the fluorophore Cy5 modified on the surface. The data characterizing the nanoparticles were measured by Luna Nanotech Inc. as well. The molar concentration of the nanoparticle stock solution was 1.36 × 10^-7^ M, and the surface charge of nanoparticles was a negative potential of -4.19 ± 0.22 mV.

### Analyzed the number of Cy5 molecules per gold nanoparticle

5 μL of the stock solution with **Cy5@Au NPs** were completely digested by 3 mL of aqua regia at mild boiling temperature. The solution was evaporated to 1 mL and cooled to room temperature. Subsequently, the sample was diluted to 3% HNO_3_ by Milli Q H_2_O, and then the amount of Au (*m*) was analyzed by inductively coupled plasma mass spectrometry (ICP-MS). Quantification was carried out by external five-point calibration. The molar concentration (*c_Au NPs_*) of **Cy5@Au NPs** is calculated as follows (*ρ*: density of Au; *r*: the radius of Au core; *N*: molar number):


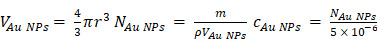


The fluorescence intensity method was used to determine the molar concentration (*c_Cy5_*) of Cy5 in the stock solution of **Cy5@Au NPs**. Quantification was carried out by external five-point calibration. The number (*R*) of Cy5 molecules per gold nanoparticles is calculated as follows:


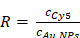


### Cell culture

HeLa cells were generously provided by the lab of Dr. Carolyn M. Price at the University of Cincinnati. The cells were cultured in DMEM containing 10% FBS, 100 units/mL of penicillin, and 100 units/mL of streptomycin in a 5% CO_2_ cell incubator (Thermo Fisher Scientific, USA) with 100% humidity at 37 °C.

### SIM imaging

All cell imaging experiments were performed with structured illumination microscopy (N-SIM, Nikon, Tokyo, Japan), a 3D-SIM equipped with an Apochromat 100×/1.49 numerical aperture oil-immersion objective lens and solid-state lasers (488 nm, 561 nm, 640 nm, the output powers at the fiber end: 15 mW). Raw SIM images (containing nine images: three phases and three angles, 70 ns exposure time per image) were reconstructed and processed with NIS-Elements AR Analysis. SIM frames were deliberately spaced at 3-s or 5-s intervals according to the purpose of each experiment. Confocal imaging was performed on the same machine (640 nm, 70 ns, 20% power). All of Pearson's colocalization coefficients (PCC) were analyzed and quantified in the open-source software CellProfiler 54.

**Cy5@Au NPs** and commercial tags were prepared with DMEM with 10% FBS, followed by staining with cells in a cell incubator. Super-resolution images were obtained with the N-SIM, which was equipped with a CMOS camera (Hamamatsu, Japan). The green channel images were excited by a 488 nm laser. The red channel images were excited by a 561 nm laser. The far-red channel images were excited by a 640 nm laser. The magenta color in images is not the natural wavelength-color, but a false color of far-red.

To dynamically track lysosomes, **Cy5@Au NPs** with an excitation wavelength of 640 nm were imaged every 5 s.

### Cell viability test

Cell cytotoxicity tests were performed using a Cell Counting Kit-8 (CCK-8, Dojindo Molecular Technologies, Inc., Japan). Once HeLa cells were planted in a 96-well plate, cell density reached at least 10,000/well, and different concentrations of **Cy5@Au NPs** were added to the wells and placed in the incubator for 24 h. After 10 μL of CCK-8 solution was added to each well, the culture plate was incubated for 2 h. Absorbance at 450 nm was determined with the Synergy Mx microplate reader (BioTek Instruments, Inc., USA).

## Results and Discussion

### Characterization data and cytotoxicity

**Figure 1:**
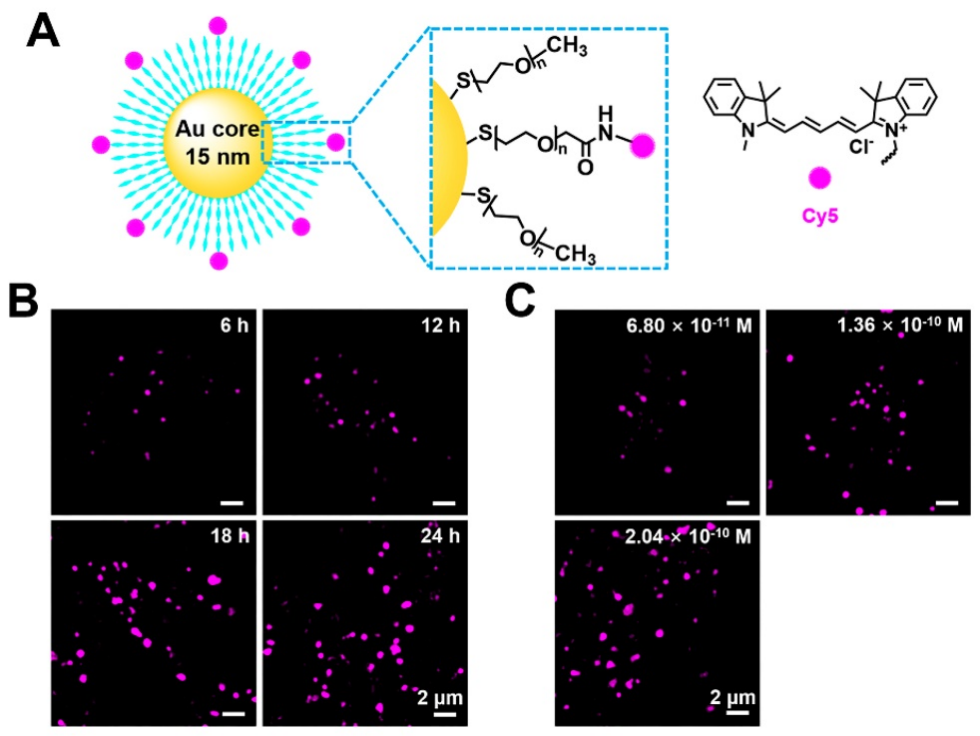
(A) Schematic illustration of the compositions of a **Cy5@Au NP**. (B) SIM images of HeLa cells stained by **Cy5@Au NPs** (1.36 × 10^-10^ M) for different treatment periods. (C) SIM images of HeLa cells stained by **Cy5@Au NPs** in different concentrations for 24 h. The dilution ratios between original nanoparticles solution and DMEM (with FBS) were 1:2000, 1:1000, and 3:2000 and the corresponding molar concentrations of nanoparticles were 6.8 × 10^-11^ M, 1.36 × 10^-10^ M, and 2.04 × 10^-10^ M, respectively.

As a cell probe, **Cy5@Au NPs** offer low cytotoxicity that is crucial for imaging. To determine their cytotoxicity, HeLa cells were seeded in a 96-well plate for 24 h and later treated with different concentrations of nanoparticles. After 24 h of the treatment of **Cy5@Au NPs**, the CCK-8 was incubated with the cells for 2 h, and the optical density of the plate was obtained. As shown in Figure S1, the cell viabilities of the concentrations tested were more than 95%, which marked a low cytotoxicity for **Cy5@Au NPs** in relation to HeLa cells and indicated the safety of using them as probes for imaging. The **Cy5@Au NPs** synthesized by Luna Nanotech contained three parts: a gold core, a biocompatible polyethylene glycol (PEG, 5 kDa) spacer, and the fluorescent dye Cy5 (Figure 1A). According to data provided by the manufacturer Luna Nanotech, the diameter of the gold core was 15 nm, and the average hydrodynamic size of the **Cy5@Au NPs** determined by dynamic light scattering in PBS buffer was 53.39 nm. To stabilize the nanoparticles against charge-induced aggregation, most PEG spacers were conjugated with methoxy to their terminal ends as backfill, and the dye-to-backfill ratio was 3:17. The number of Cy5 on one particle was calculated to be approximately 145. Under 640-nm laser irradiation, an emission between 650 nm and 750 nm for Cy5 was excited, for an emission wavelength that peaked at 670 nm (Figure S2 and S3).

### Time-dependent and concentration-dependent cellular uptake

To explore **Cy5@Au NP** as a potential probe in cells, the cellular internalization of the particles was investigated. Considering that endocytosis is the internalized mechanism of large particles in cells, an interval of 6 h was set as the treatment period. The human cervix carcinoma cell line HeLa was incubated with the particles and imaged under SIM. As shown in Figure 1B, punctate fluorescence was observed. Over time, the punctate fluorescent intensity increased and became stable at 24 h, after which different concentrations of **Cy5@Au NPs** internalized by cells were studied for 24 h. The fluorescence intensity at a concentration of 6.8 × 10^-11^ M was too weak to be visible, whereas the intensity at a concentration of 2.04 × 10^-10^ M did not considerably boost the intensity at a concentration of 1.36 × 10^-10^ M (Figure 1C). Therefore, the concentration of 1.36 × 10^-10^ M and treatment time of 24 h were applied for the super-resolution imaging in the experiments that followed.

### Super-resolution imaging of lysosomes

**Figure 2:**
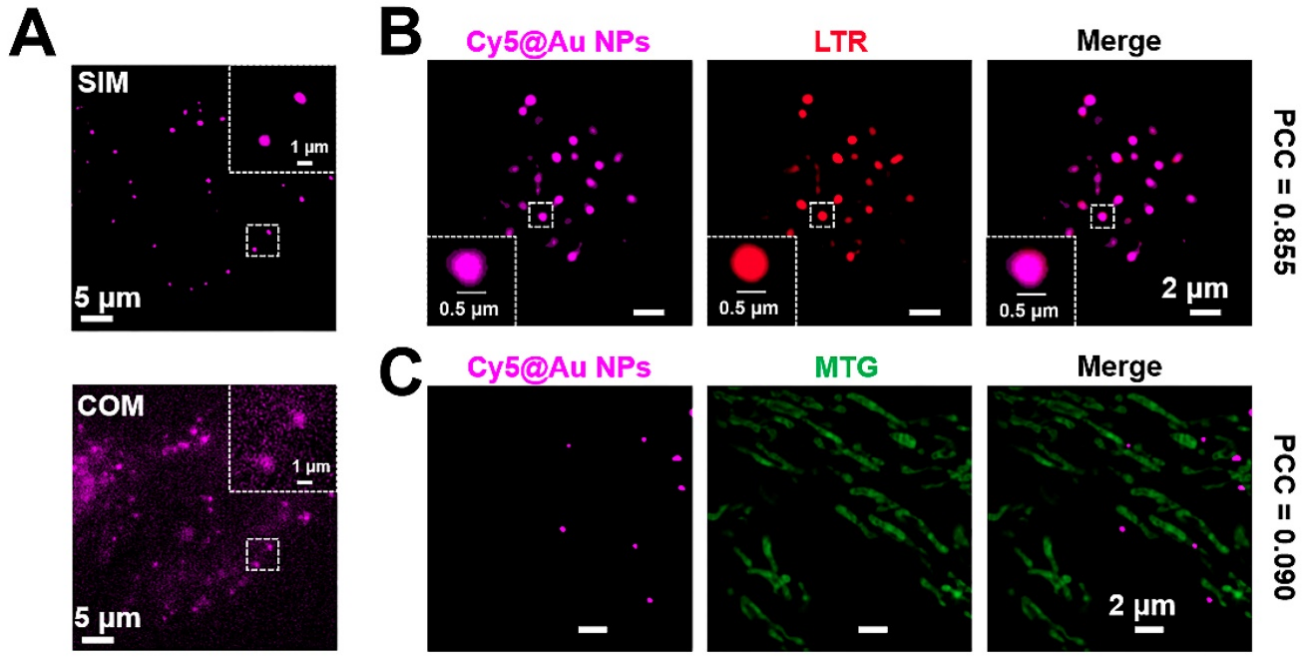
(A) Images of COM and SIM. (B) SIM images of a colocalization experiment with HeLa cells costained with **Cy5@Au NPs** and LTR. (C) SIM images of a colocalization experiment with HeLa cells costained with **Cy5@Au NPs** and MTG.

Confocal optical microscopy (COM) has been widely used for cell imaging. To compare the imaging quality of COM and SIM, HeLa cells treated with **Cy5@Au NPs** were imaged under both types of microscopy, which revealed that the SIM image had a lower fluorescence background and a clearer punctate fluorescence (Figure 2A). Moreover, the full width at half-maximum (FWHM) values of SIM are smaller than the values of COM, demonstrating the advantage using SIM (Figure S4) 55, 56. To ensure the localization of the punctate fluorescence in cells, a colocalization assay was performed. After 24 h of treatment with **Cy5@Au NPs**, the lysosomes and mitochondria of HeLa cells were respectively stained with the commercial dyes LTR and MTG for SIM imaging. As shown in Figure 2B, a high overlap emerged in the fluorescence of **Cy5@Au NPs** and LTR, with a PCC of 0.855. By contrast, the PCC of **Cy5@Au NPs** and MTG, at only 0.090, indicated little overlap (Figure 2C). The lysosomal colocalization assays of **Cy5@Au NPs** were also performed with LTG and LAMP1-mGFP, and high PCC values were obtained (Figure S5 and S6). However, because of low transfection efficiency, only a few of cells with fluorescence of LAMP1-mGFP were found. The results thus imply that **Cy5@Au NPs** distributed in lysosomes specifically. Moreover, the dye Cy5, when incubated with HeLa cells for 24 h, showed a PCC of 0.624 with the commercial lysosomal dye (Figure S7), which suggests that the nanoparticles helped the dye to localize specifically in lysosomes.

### Cy5@Au NPs in lysosomes during microenvironment change

**Figure 3:**
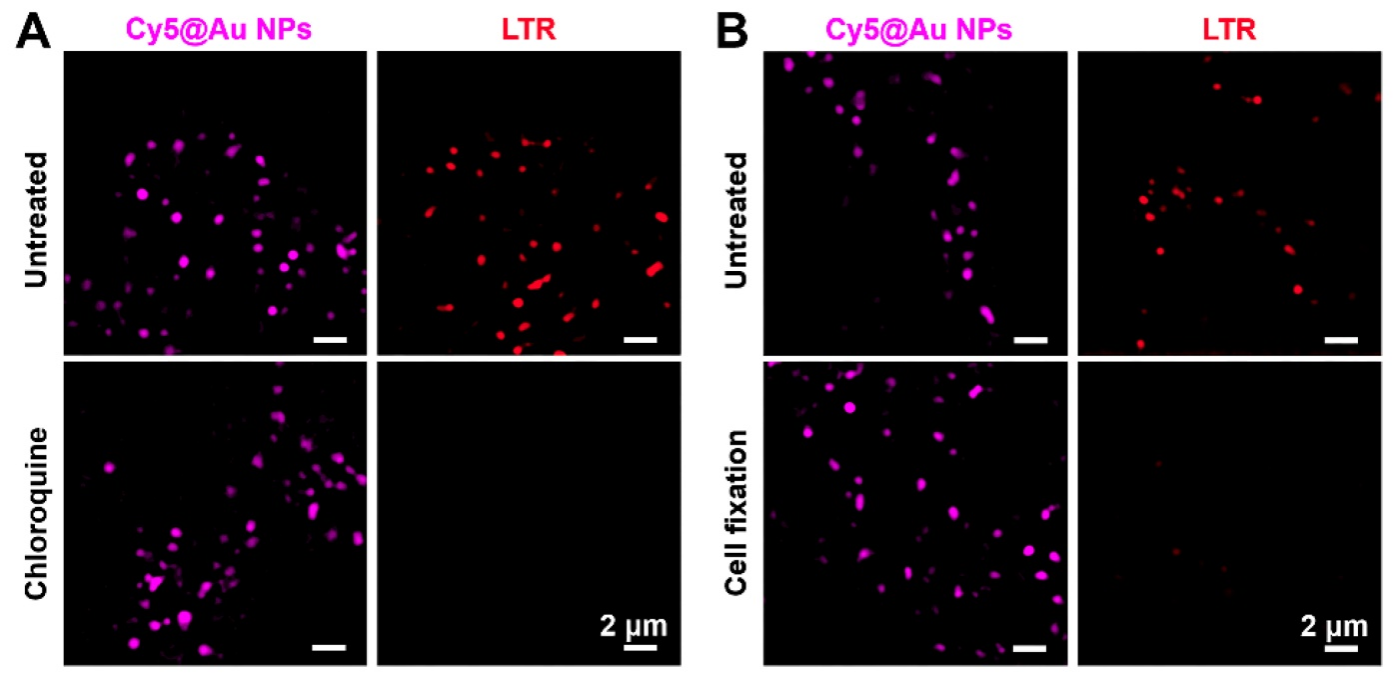
(A) SIM images of untreated and chloroquine-treated HeLa cells stained with **Cy5@Au NPs** and LTR, respectively. (B) SIM images of untreated and 4% paraformaldehyde-treated HeLa cells (i.e., for cell fixation) stained with **Cy5@Au NPs** and LTR, respectively.

The pH value of the microenvironment for lysosomes ranges from 4.5 to 5.0. Commercial dyes (e.g., LTG and LTR), consisting of a fluorophore and a weak base group, can freely permeate cell membranes, and once the weak base group protonates in the acidic microenvironment, LysoTrackers can be trapped in lysosomes. By the same token, when the lysosomal pH changes, the localization of deprotonated LysoTrackers lose track of their target 41, 42. To determine whether **Cy5@Au NPs** enter lysosomes by endocytosis and remain there despite fluctuations in pH, the antimalarial drug chloroquine, a cell-permeable base, was used to stimulate living cells to raise the lysosomal pH. After treatment with LTR and LTG particles, HeLa cells were incubated with chloroquine in a concentration of 100 μM for 30 min. Before treatment with chloroquine, bright fluorescence from particles and commercial lysosomal dyes was observed; after treatment, the fluorescence of particles remained in cells, although the signals of LTR and LTG had faded (Figure 3A and S8A).

### Cy5@Au NPs in lysosomes during LMP

The commercial dyes LTR and LTG are easily washed out when cells are subjected to cell fixation. After cells were fixed in our study, the membrane of lysosomes underwent permeabilization, and the molecular LTR and LTG escaped. As shown in Figures 3B and S8B, the fluorescence signals of LTR and LTG even disappeared in the fixed cells. By contrast, **Cy5@Au NPs** overcame that limitation, and a bright punctate fluorescence of **Cy5@Au NPs** for lysosomal staining was achieved in the fixed cells. Ultimately, LMP failed to eliminate nanoparticles from the lysosomes. The results thus suggest that **Cy5@Au NPs** can remain in lysosomes no matter how the lysosomal microenvironment and its membrane change.

### Anti-photobleaching

**Figure 4:**
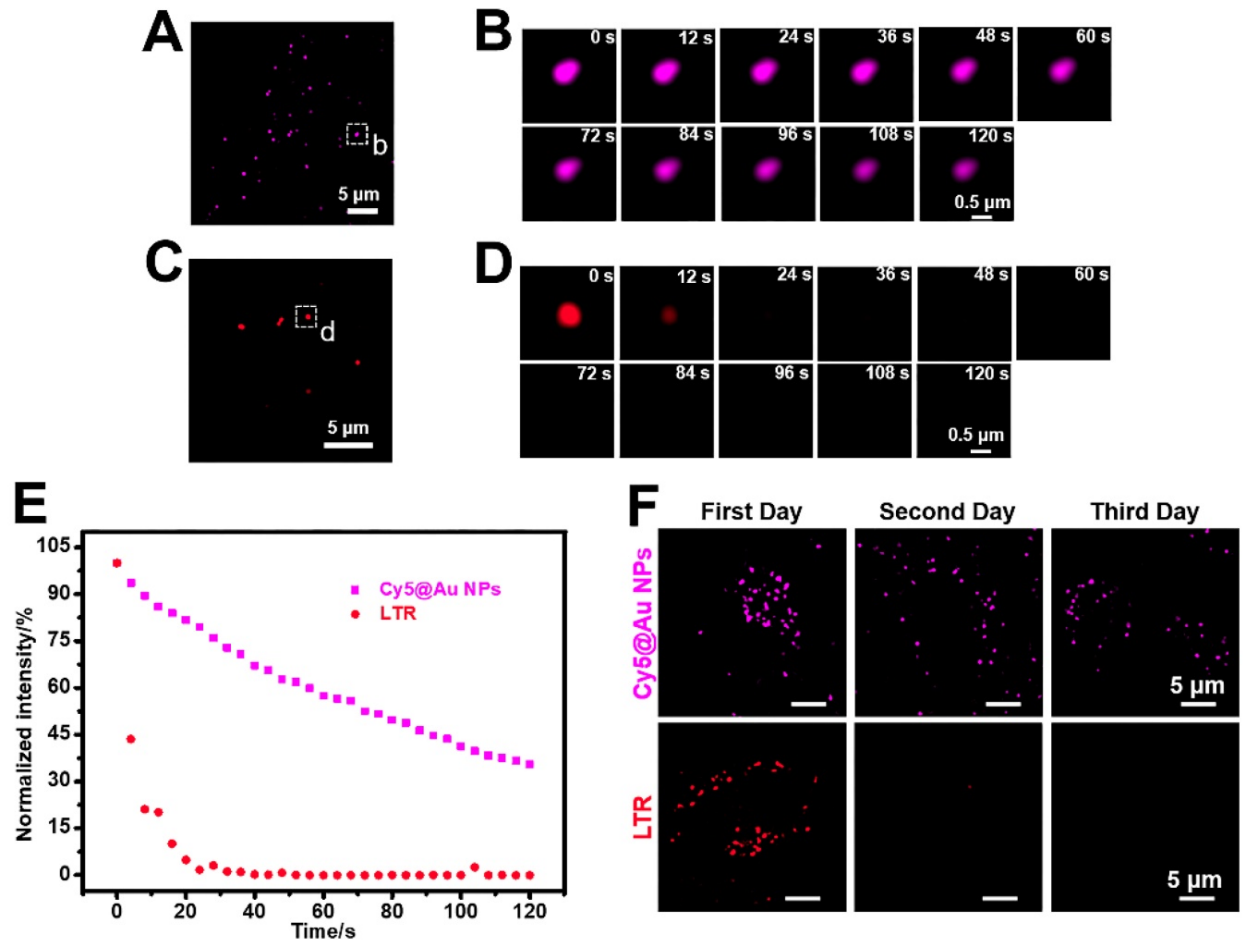
(A) SIM image of HeLa cells stained with **Cy5@Au NPs** before photobleaching. (B) Magnified images of the dashed box in (A) during photobleaching. (C) SIM image of HeLa cells stained with LTR before photobleaching. (D) Magnified images of the dashed box in (C) during photobleaching. (E) Normalized intensity during photobleaching of **Cy5@Au NPs** and LTR. (F) SIM images of HeLa cells stained with **Cy5@Au NPs** and LTR for 3 d.

For all subcellular probes, photostability is crucial for imaging. The photobleaching assays of **Cy5@Au NPs**, LTG, LTR, and LAMP1-mGFP were performed under laser irradiation with 100% power (15 mW). After 80 s of irradiation, the fluorescence intensity of LTR was entirely bleached (Figure 4C-4E), and remained at 18% for LTG, 30% for LAMP1-mGFP, and 50% for **Cy5@Au NPs** (Figure S9). As irradiation continued, the fluorescence intensity of **Cy5@Au NPs** decreased but remained at 35% after 120 s of irradiation (Figure 4A, 4B, and 4E). The results showed that **Cy5@Au NPs** displayed excellent anti-photobleaching ability.

### Long-term lysosomal labelling

To understand the particulars of lysosomal functions, the long-term labelling ability of lysosomal probes is pivotal 37, 57, hence our investigation of that ability in **Cy5@Au NPs**. After 24 h of treatment with **Cy5@Au NPs** (i.e., the first day), the fluorescence of HeLa cells was captured by SIM. Next, the petri dish was washed with PBS buffer 3 times and kept in the incubator for another 24 h. Given the space of the dish and the rate of cell proliferation, the experiment was performed for 3 d. For contrast, LTR and LTG were tested in the same way. As shown in Figures 4F and S10, the fluorescence signals of LTR and LTG nearly disappeared on the second day. The fluorescence of **Cy5@Au NPs** was observable on the third day, although its intensity had decreased over time. In sum, the assay exhibited the long-term (i.e., >3 d) lysosomal labelling ability of **Cy5@Au NPs**.

### Kiss-and-run process, fusion, and fission of lysosomes

**Figure 5:**
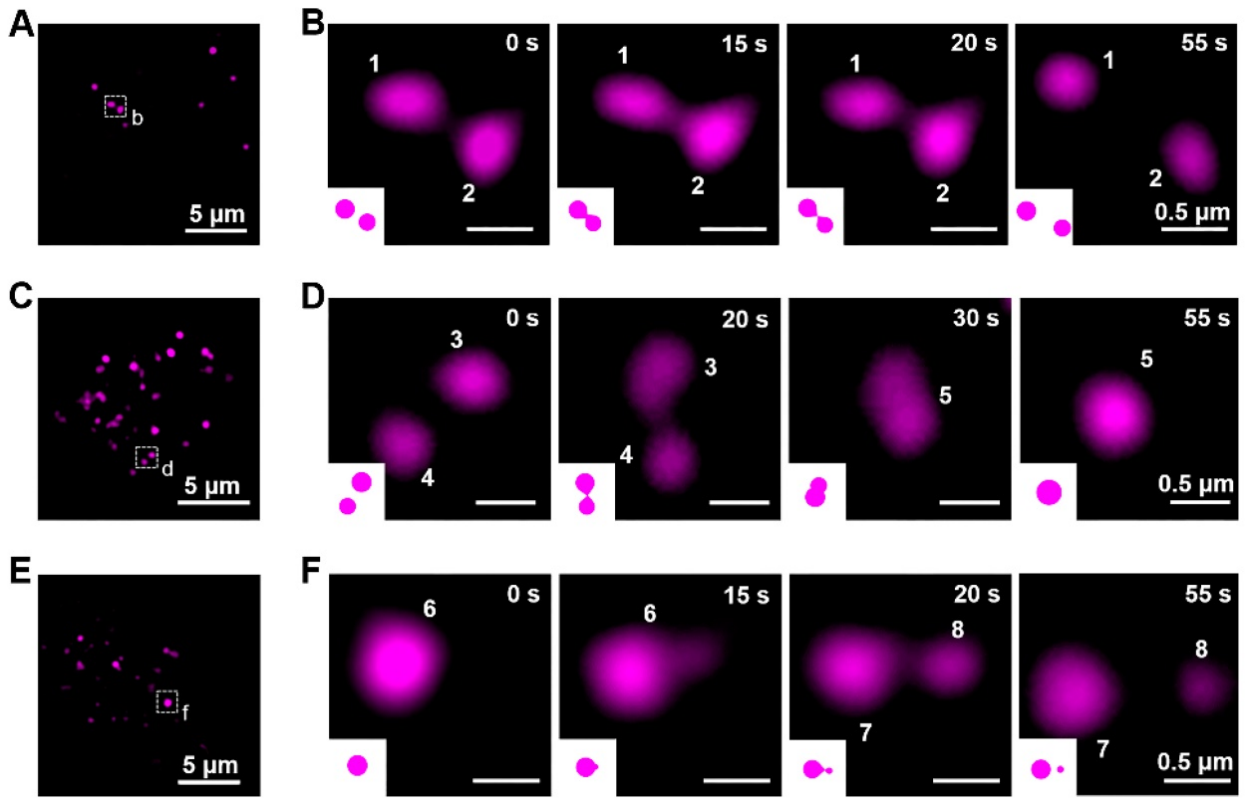
The dynamics of lysosomes in living cells. (A) SIM image of HeLa cells stained with **Cy5@Au NPs** before kiss-and-run process. (B) Magnified images of the dashed box in (A) during kiss-and-run process. (C) SIM image of HeLa cells stained with **Cy5@Au NPs** before fusion. (D) Magnified images of the dashed box in (C) during fusion. (E) SIM image of HeLa cells stained with **Cy5@Au NPs** before fission. (F) Magnified images of the dashed box in (E) during fission.

To exercise their cellular functions, lysosomes have to move and interact with each other 58-61. Using the lysosome probe **Cy5@ Au NPs**, the fluorescent dynamics of lysosomes were captured by SIM, and their lysosome-lysosome interactions were categorized as kiss-and-run process, fusion, and fission. The kiss-and-run process was recorded first. As shown in Figures 5A, 5B, and S11A as well as in Video S1, two fluorescence lysosomes, labeled 1 and 2, kissed within 15 s and ran away at the 20-s mark. Kiss-and-fusion, as a fusion event, is depicted in Figures 5C, 5D, and S11B as well as in Video S2; therein, two fluorescence lysosomes, labeled 3 and 4, kissed and fused within 10 s to form a hybrid lysosome, labeled 5. Beyond that, during fission, lysosomes can self-divide into multiple lysosomes; thus, for fission, a large lysosome, labeled 6, emerged at first and split into two other lysosomes, labeled 7 and 8, within 5 s and thereafter separated (Figure 5E, 5F, and S11C, Video S3).

### Imaging mitophagy in HeLa cells

**Figure 6:**
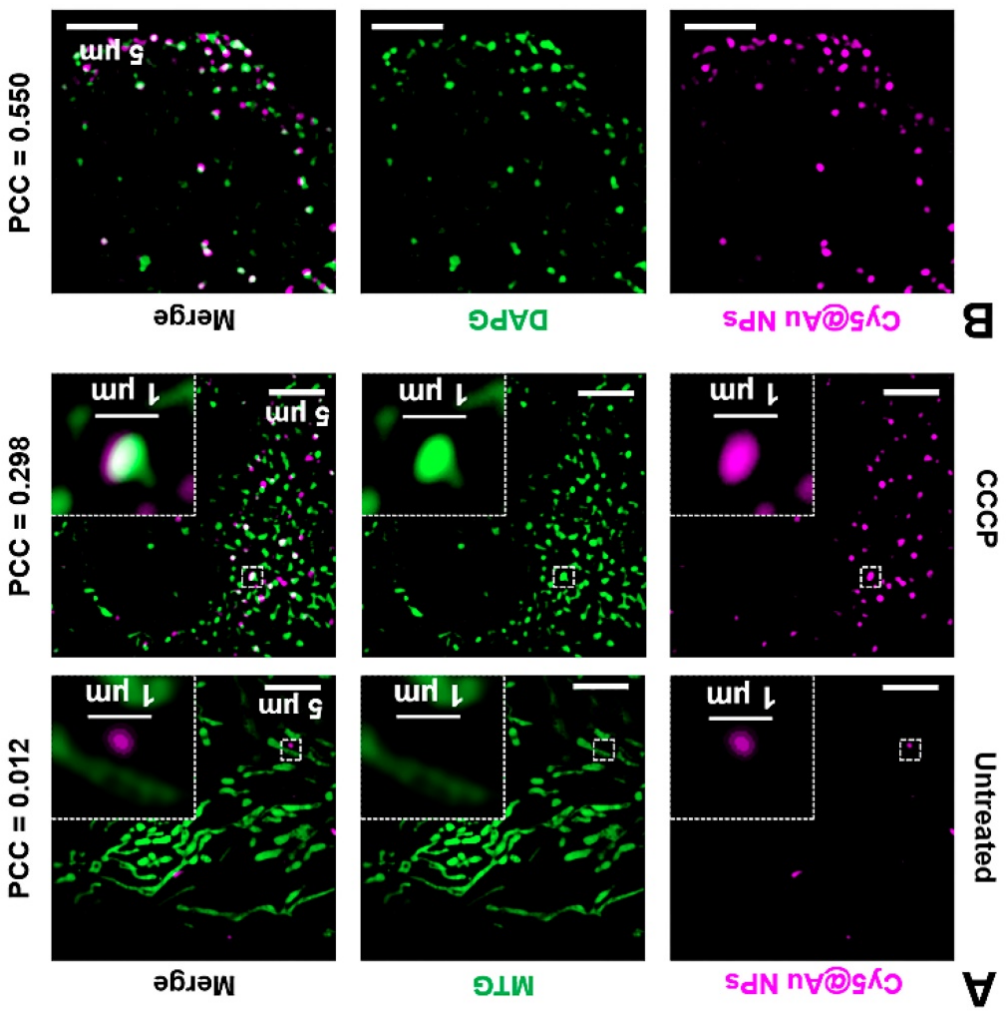
(A) SIM images of untreated and CCCP-treated HeLa cells stained with **Cy5@Au NPs** and MTG. (B) SIM images of CCCP-treated HeLa cells stained with **Cy5@Au NPs** and DAPGreen (DAPG).

As a vital organelle, lysosomes are involved in many important cellular functions, including autophagy 62, 63, during which a cell consumes itself and is decomposed by lysosomes. Mitophagy is a well-known type of autophagy. Mitochondria are important sites for cellular respiration that provide energy to cells. When mitochondria are damaged, the cell initiates mitophagy in order to recycle the damaged mitochondria 64-66. CCCP, a de-coupler of oxidative phosphorylation that abolishes the mitochondrial membrane proton gradient, can induce mitochondrial damage 10, 67. In our study, after staining MTG and **Cy5@Au NPs**, HeLa cells were thus treated with 10 μM of CCCP for 12 h to damage the mitochondria. Investigated under SIM, the morphologies of most mitochondria changed from filamentous to spherical (Figure 6A), thereby indicating that the mitochondria had been severely damaged. Compared to the untreated cells, the number of lysosomes increased, and the lysosomes showed an increasing overlap of 0.298 with the mitochondria, indicating the fusion of lysosomes and mitochondria 68, 69. DAPGreen is a commercial fluorescent molecule for autophagosomes and autolysosomes detection, and can be used to detect autophagy 70. To confirm the occurrence of autophagy, DAPGreen was incubated with the CCCP-treated HeLa cells for 30 min. A symbolic fluorescent signal of DAPGreen indicating autophagy appeared in the cells (Figure 6B), and some of the DAPGreen fluorescence fused with the lysosomes (PCC 0.550), which implies that autolysosomes have formed and that autophagy has occurred.

## Conclusion

The fluorescent gold nanoparticles were used as lysosomal probes for the super-resolution imaging in living cells. Compared with commercial lysosomal dyes, **Cy5@Au NPs** exhibited stronger tolerance in lysosomes during various treatments, and changes in the lysosomal microenvironment and LMP did not cause **Cy5@Au NPs** to lose track of their targets. **Cy5@Au NPs** demonstrated an excellent anti-photobleaching ability, and a long-term labelling assay revealed that they could stain lysosomes more than 3 d. Moreover, the particles exhibited low cytotoxicity to HeLa cells. Last, biological events such as the kiss-and-run process, fusion, fission, and mitophagy were recorded with the fluorescent **Cy5@Au NPs** under SIM. All of those results indicate that **Cy5@Au NPs** can be a useful tool for visualizing lysosomes in super-resolution. For the simple modification of fluorophores on the surface, nanoparticles with different fluorophores can meet the requirements of various experiments. Beyond that, autophagy has recently been characterized as a pathway for drug-resistant cells to escape death, and the phagocytosis of drug-damaged organelles was shown to prevent the initiation of apoptosis 71-73. As lysosomal probes, **Cy5@Au NPs** can thus be further used to observe the difference of autophagy between cancer cells and corresponding drug-resistant cells in the future.

## Abbreviations

STORM: stochastic optical reconstruction microscopy
STED: stimulated emission depletion
SIM: structured illumination microscopy
LTG: LysoTracker Green
LTR: LysoTracker Red
LMP: lysosomal membrane permeabilization
PEG: polyethylene glycol
Cy5: cyanine 5
CCCP: carbonyl cyanide m-chlorophenylhydrazone
MTG: MitoTracker Green FM
FBS: fetal bovine serum
DMEM: Dulbecco's modified Eagle's medium
PBS: phosphate-buffered saline
PCC: Pearson's colocalization coefficients
COM: confocal optical microscopy
