# Supplementary material for: Super-resolution observation of lysosomal dynamics with fluorescent gold nanoparticles
Source: Theranostics. 2020 May 15;10(13):6072–81. doi: 10.7150/thno.42134 (PMC7254985; doi:10.7150/thno.42134)
Supplement: Supplementary file 1 — Supplementary figures. [file thnov10p6072s1.pdf]

Supplementary Material

Super-resolution observation of lysosomal dynamics with fluorescent gold nanoparticles

Kangqiang Qiu[1,2], Yang Du[2,3], Jiyan Liu[3], Jun-Lin Guan[2], Hui Chao[1]*, Jiajie Diao[2]*

1. MOE Key Laboratory of Bioinorganic and Synthetic Chemistry, School of Chemistry, Sun Yat-Sen University, Guangzhou 510275, China.
2. Department of Cancer Biology, University of Cincinnati College of Medicine, Cincinnati, OH 45267, USA.
3. Department of Biotherapy, Cancer Center, State Key Laboratory of Biotherapy, West China Hospital, West China Medical School, Sichuan University, Chengdu 610041, China.

* ceschh@mail.sysu.edu.cn (H.C.); jiajie.diao@uc.edu (J. D.).

Table of content

Figure S1 Cytotoxicity of **Cy5@Au NPs**……..S2

Figure S2 Characterization data of **Cy5@Au NPs**...S3

Figure S3 Characterization parameters of **Cy5@Au NPs**... S4

Figure S4 Fluorescence intensity profiles of SIM and COM images...S5

Figure S5 Colocalization images of **Cy5@Au NPs** and LTG..S6

Figure S6 Colocalization images of **Cy5@Au NPs** and LAMP1-mGFP..................................S7

Figure S7 Colocalization images of Cy5 dye and LTG and MTG...S8

Figure S8 Images of chloroquine-treated and fixed cells stained with LTG.............................S9

Figure S9 Photobleaching of LTG or LAMP1-mGFP..S10

Figure S10 SIM images of HeLa cells stained with LTG for 3 d..S11

Figure S11 Intensities of **Cy5@Au NPs** during different processes......................................S12

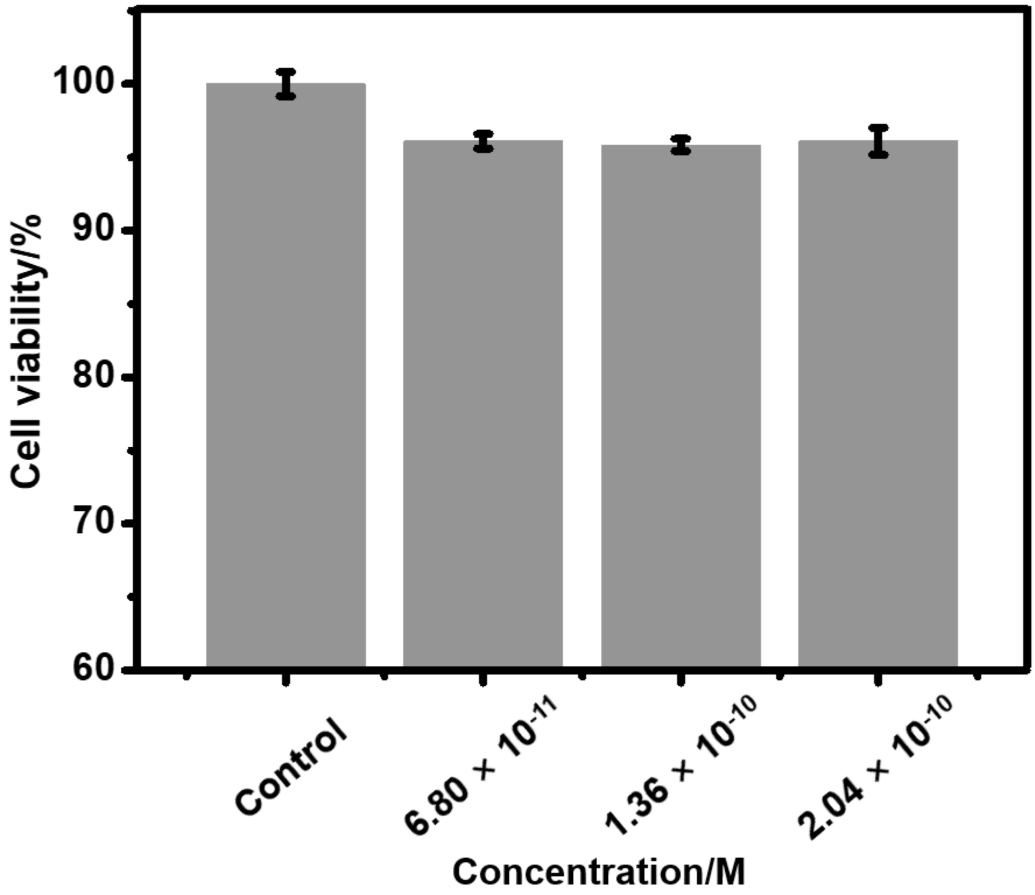

Figure S1. HeLa cells viability treated with different concentration of **Cy5@Au NPs** for 24 h via CCK-8 assay.

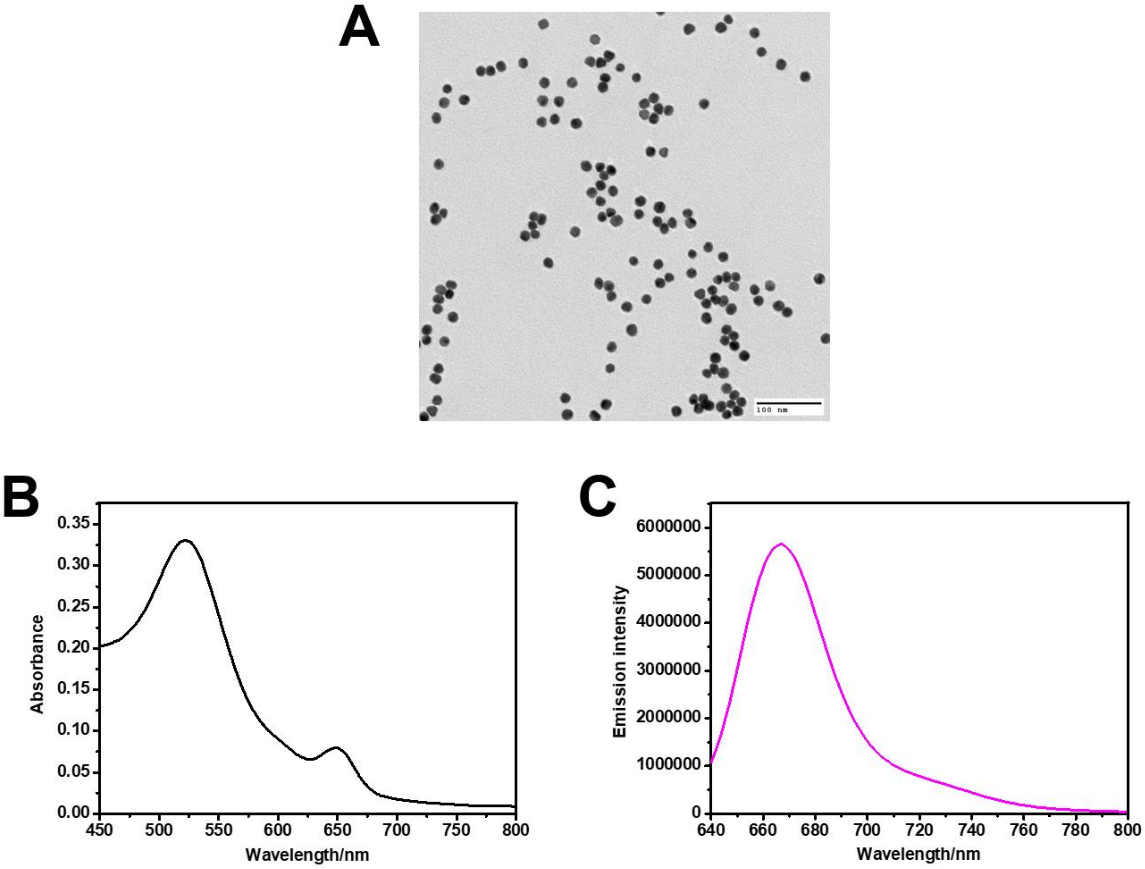

Figure S2. Characterization data of **Cy5@Au NPs** provided by Luna nanotech. (A) TEM image of the particles (scale bar: 100 nm); (B) Absorption and (C) fluorescence spectra of **Cy5@Au NPs**.

Parameter	Value
Core Diameter:	15 nm
Z-Average (DLS):	53.39 nm
PDI (DLS):	0.134
Z-Potential[1]:	-4.19(+/-0.22) mV
Nanoparticle OD:	50
Nanoparticle Molar Conc.:	1.36×10^{-7} M
Solution Volume:	0.4 mL
Dye:	Cy5
PEG-Dye / mPEG Backfill:	15% / 85%
PEG-Dye Size	5 kDa
mPEG Backfill Size	5 kDa
Shelf Life:	1 year
Storage Buffer:	1x PBS

[1] Z-Potential measurements were performed in 10mM HEPES, 1 mM NaCl, pH 7.4 buffer.

Figure S3. Characterization parameters of **Cy5@Au NPs** provided by Luna nanotech.

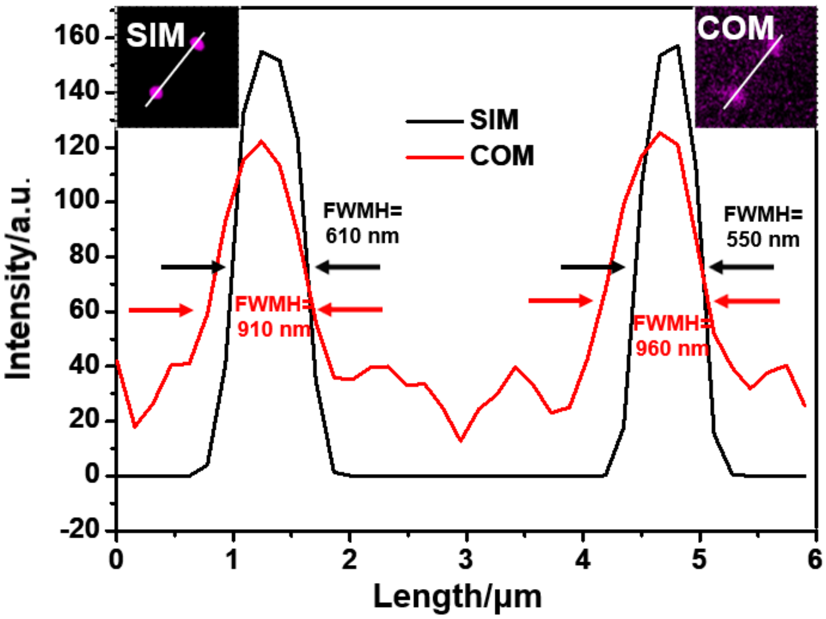

Figure S4. SIM and COM images and their corresponding fluorescence intensity profiles and calculated FWMH.

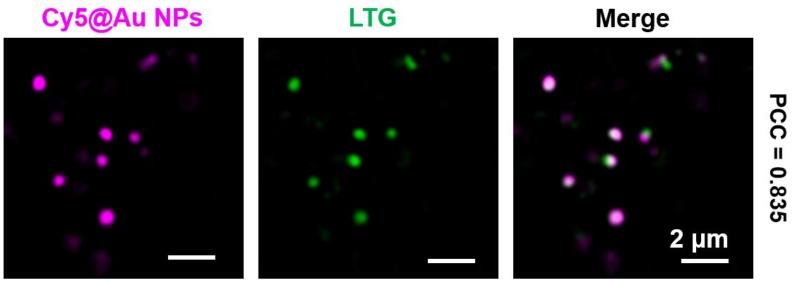

Figure S5. SIM images of a colocalization experiment with HeLa cells costained with **Cy5@Au NPs** and LTG.

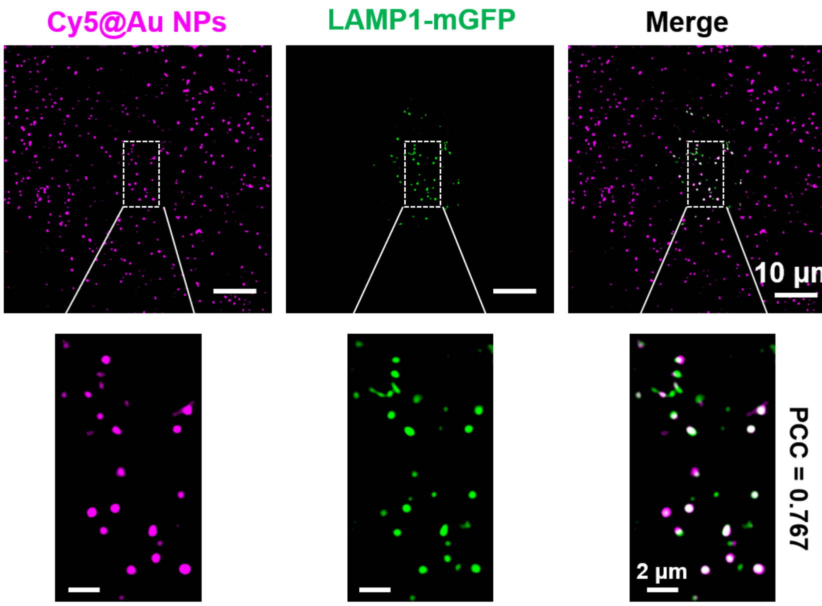

Figure S6. SIM images of a colocalization experiment with HeLa cells costained with **Cy5@Au NPs** and LAMP1-mGFP.

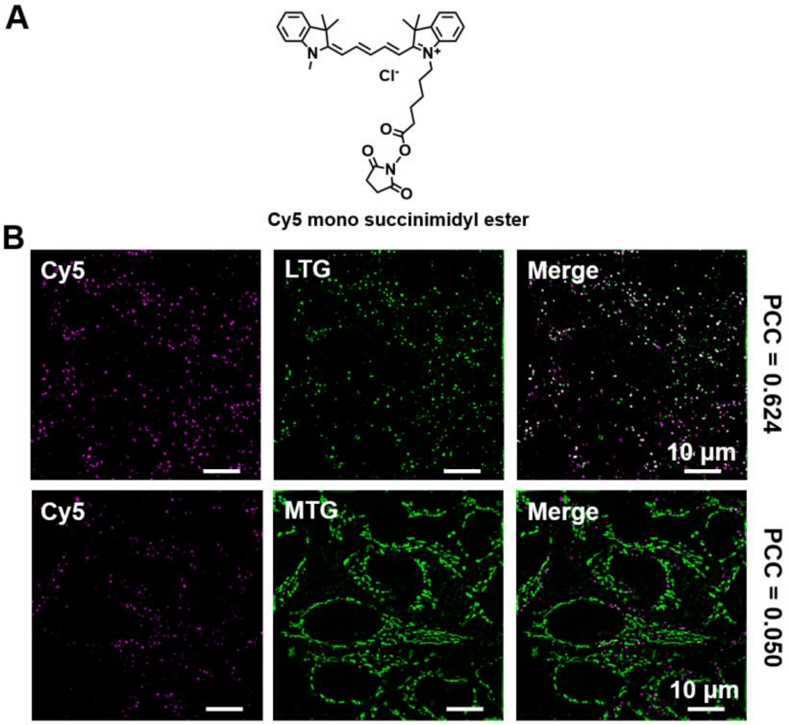

Figure S7. (A) The chemical structure of Cy5 for the experiment. (B) Colocalization images of HeLa cells stained by Cy5 and the commercial dyes LysoTracker Green (LTG) and MitoTracker Green (MTG).

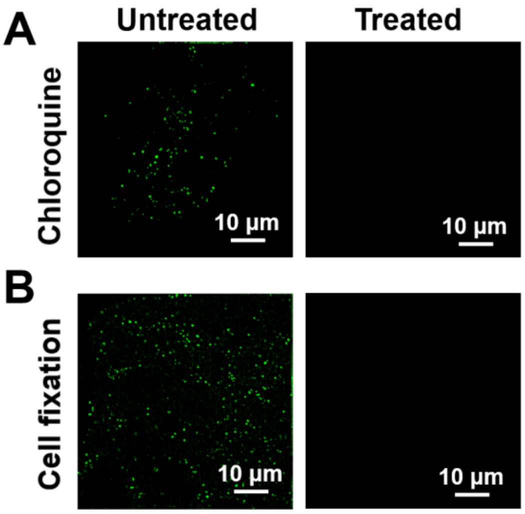

Figure S8. Images of untreated and (A) chloroquine-treated or (B) 4% paraformaldehyde-treated HeLa cells stained with LTG.

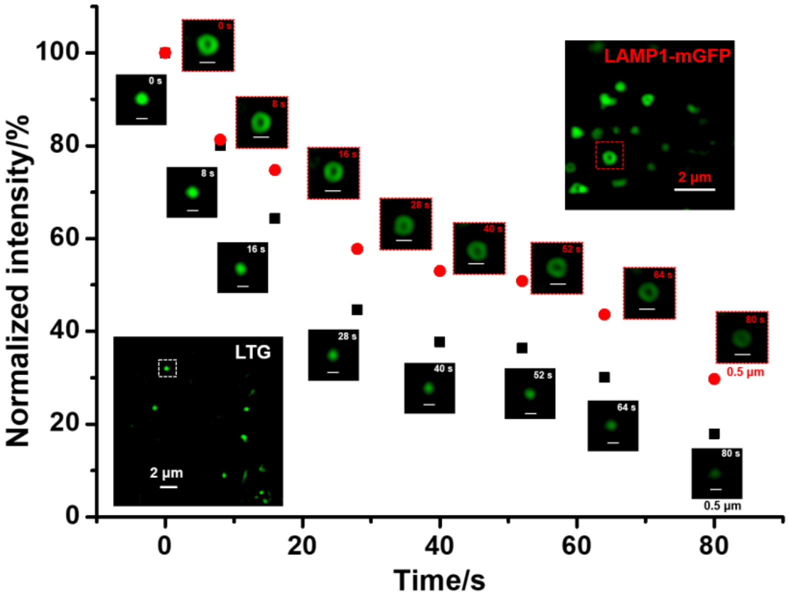

Figure S9. SIM image of HeLa cells stained with LTG or LAMP1-mGFP and their normalized intensity during photobleaching.

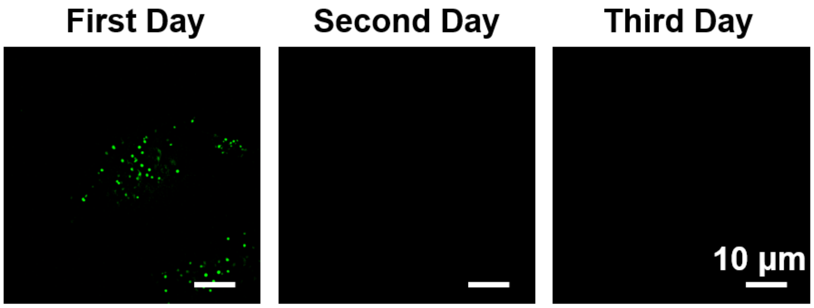

Figure S10. SIM images of HeLa cells stained with LTG for 3 d.

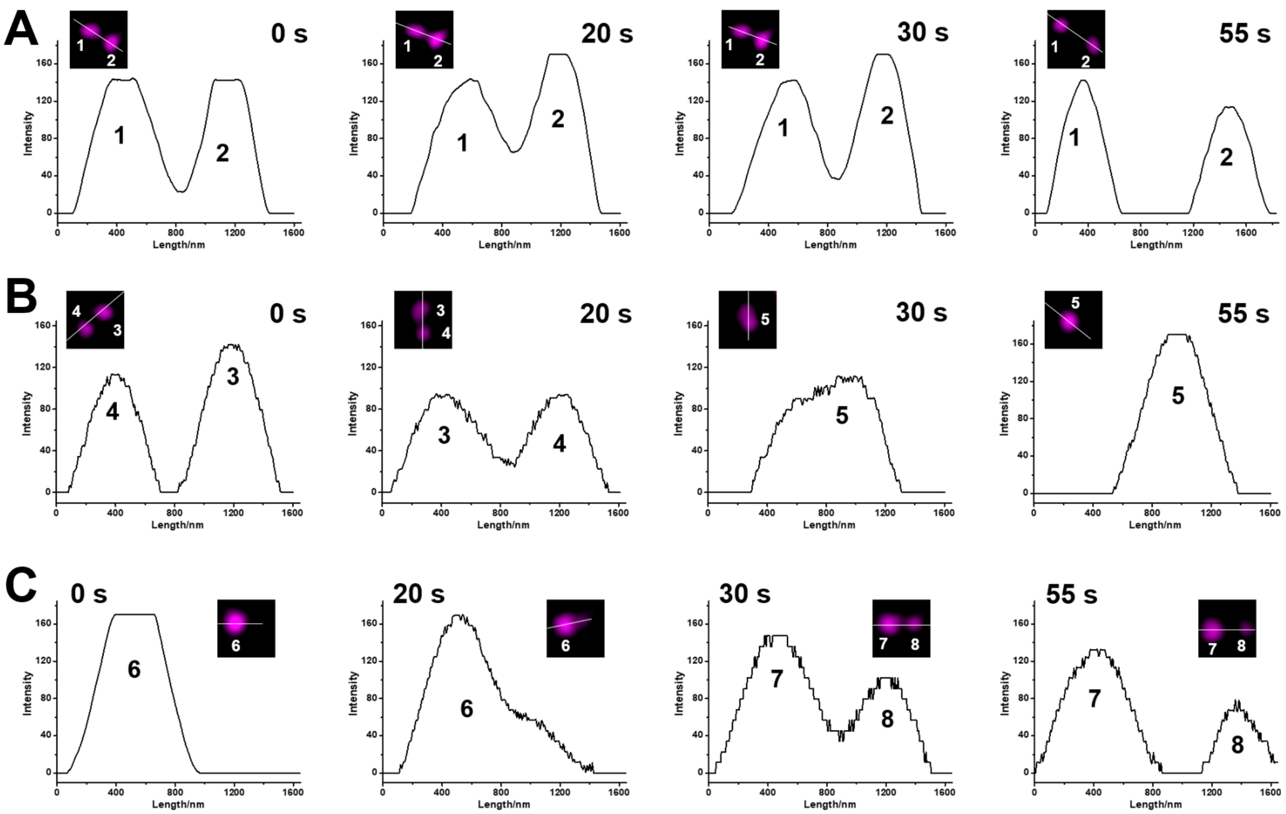

Figure S11. Intensities of **Cy5@Au NPs** on the white line in the images. (A) Kiss-and-run process. (B) Fusion. (C) Fission.
